# Finite element analysis of stress concentration between surface coated implants and non surface coated implants - An in vitro study

**DOI:** 10.4317/jced.55915

**Published:** 2019-08-01

**Authors:** Tammineedi SV. Satyanarayana, Rathika Rai, E. Subramanyam, T. Illango, Vishwabharathi Mutyala, Rajesh Akula

**Affiliations:** 1 MDS (Prosthodontics), Reader, Lenora institute of dental sciences, Rajahmundry-533103; 2 MDS (Prosthodontics), Principal,HOD Dept of prosthodontics, Thaimoogam bigai Dental college, Chennai; 3 MDS (Prosthodontics), Ex HOD Dept of prosthodontics, Thaimoogam bigai Dental college, Chennai; 4 MDS (Prosthodontics), Ex Prof. Dept of prosthodontics, Thaimoogam bigai Dental college, Chennai; 5 SBS Ayurvedic Medical College, Mundurgi; 6 MDS (Prosthodontics), Dental officer, Ex-servicemen Contributory Health Scheme, Srikakulam

## Abstract

**Background:**

To determine qualitative comparison in stress distribution between surface coated implants and non surface coated implants using 2 different lengths and vertical, oblique, and lateral forces.

**Material and Methods:**

3 dimensional finite element study was carried out at first molar site with 4 surface coated and 4 non surface coated implants using mimic 8.11, solid edge 2004, hypermesh 9.0, and ansys12.1 software.

**Results:**

The pattern of stress distribution was almost similar between vertical and oblique loading but varied with lateral loads between surface coated and non surface coated implants. As the length of the implants increased stress concentration had no significant variation between surface coated and non surface coated implants, but had a tendency to increase at the abutment and abutment screw on all 3 forces.

**Conclusions:**

Among the surface coated and non surface implants the pattern of stress distribution was similar signifying that surface coating of implants had no significant role in stress distribution using 3d finite element analysis and within the limitations of this study.

** Key words:**Surface coating, non surface coating, implants, stress and bone.

## Introduction

Significant progress has been made in the clinical use of oral and maxillofacial implants over the past three decades. Statistics on the use of dental implants reveals about 100,000 to 300,00 dental implants are placed per year as the aging population increases larger number of individuals are being defined as partially edentulous, using dental implants as a recent standard care (1).

Biomechanical optimization is an important objective in the design of dental implants. Although the success rates of some implant systems have been high, implant failures; do occur even operated by a professional implantologist (2,3). The failure is in part due to the occlusal forces of various magnitudes and directions that the dental implants sustain, some of which can be very large (4). One of the effective ways to maintain excellent clinical performance is to use a biomechanically optimized implant that provides a health stress strain level required for normal bone resorption and deposition processes at the implant site. Most efforts have been directed at optimizing implant geometry in order to maintain a beneficial stress level at the bone-implant interface. The effects of various parameters such as load direction and bone quality on the performance of a dental implant are very important. A better understanding of these effects will lead to a significant improvement in the design of dental implants (5-7).

A key factor for the success or failure of a dental implant is the manner in which stresses are transferred to the surrounding bone (7). Vertical and transverse loads from mastication induce axial forces and bending moments and result in stress gradients in the implant as well as in the bone. Forces and moments transferred from implants to the surrounding bone depend on the type of loading, the bone implant interface, implant geometry, the prosthesis type, and the quantity and quality of the surrounding bone (5). The continuing search for “osseoattractive” implants is leading to surface modification involving biological molecules by attaching or releasing powerful cytokines and growth factors, desired cell and tissue responses may be obtained. Using even a simple delivery system introduction of bone morphogenetic protein at the tissue implant interface was shown to enhance the rate of periprosthetic bone formation.

Biological factors determine the maintenance and enhancement of implant stability hence the importance of an implant surface that favorably supports the biological bone healing process is of paramount importance the cumulative results of which is a significantly stronger and faster osseointegration of the implant and the effects of stress distribution in these implants . Optimizing implant geometry in order to maintain a beneficial stress level at the bone-implant interface is a complex issue (8).

Researchers can predict stress distributions in the contact area of an implant in cortical bone and around the apex of an implant in trabecular bone with Finite element analysis (FEA). FEA is an effective tool used to evaluate the biomechanical characteristics of different types of dental implants. The literature reflects that it has been widely used to model the design and functionality of dental implants and predict features of design optimization (7).

-Aims and Objectives

The purpose of this study was to arrive at a qualitative comparison in the stress concentration/distribution between the surface coated implants and non surface coated implant by using computer simulations to examine clinical situations.

Three-dimensional finite element analysis models were used to evaluate the stress distribution patterns

1. crown, abutment, inscrew, implant, soft bone, hard bone

2. Loading directions. (vertical -30n, lateral - 10n, oblique -70n)

3. Different lengths of the same implant

4. Among surface coated implants and non surface coated implants.

## Material and Methods

In the present study 3 Dimensional Finite element study was carried out at the first molar site with 8 different commercially available implants, (Out of which 4 were surface coated and other 4 were non surface coated implants) to determine the stress distribution patterns. The implants used in the study are

Surface coated implants

1) Noble biocare Replace select tapered tiU 4.3mmD 10mmL

2) Noble biocare Replace select tapered tiU 4.3mmD 13mmL

3) Zimmer Tapered screw-vent 4.1mmD 10mmL

4) Zimmer Tapered screw-vent 4.1mmD 13mmL

Non surface coated implants

1) Adin tapered 4.2mmD 10mmL

2) Adin tapered 4.2mm D 13mmL

3) Uniti tapered 4.2mmD 10mmL

4) Uniti tapered 4.2mmD 13mmL

Loads and boundary conditions: -

For all the cases the bottom portion of the cortical bone and cross-sectional faces on either side of the bone is fixed. A vertical (30 N), Horizontal (10 N) and oblique (70 N), emulating the masticatory load, periodontal force and the muscle force respectively were in turn applied to each of the above models.

Loading pattern:-

1. Vertical force of 30 N

2. Lateral force of 10N

3. Oblique force of 70N

Software details

• CT scan of the bone and crown is taken into mimics 8.11 software

• Surface data of the implant, abutment and inscrew is generated using solid edge 2004 software

• Finite element model is generated using Hypermesh 9.0 software

• Analysis was carried out using Ansys 12.1 software

Hardware details

• Intel core 2 duo processor

• 4GB ram

• 320GB hard disk

Color coding for Von-Mises Stress

Blue is minimum stress and red is the maximum in between shades are the variation of stress from minimum to maximum

Methodology.

**Figure 1:**
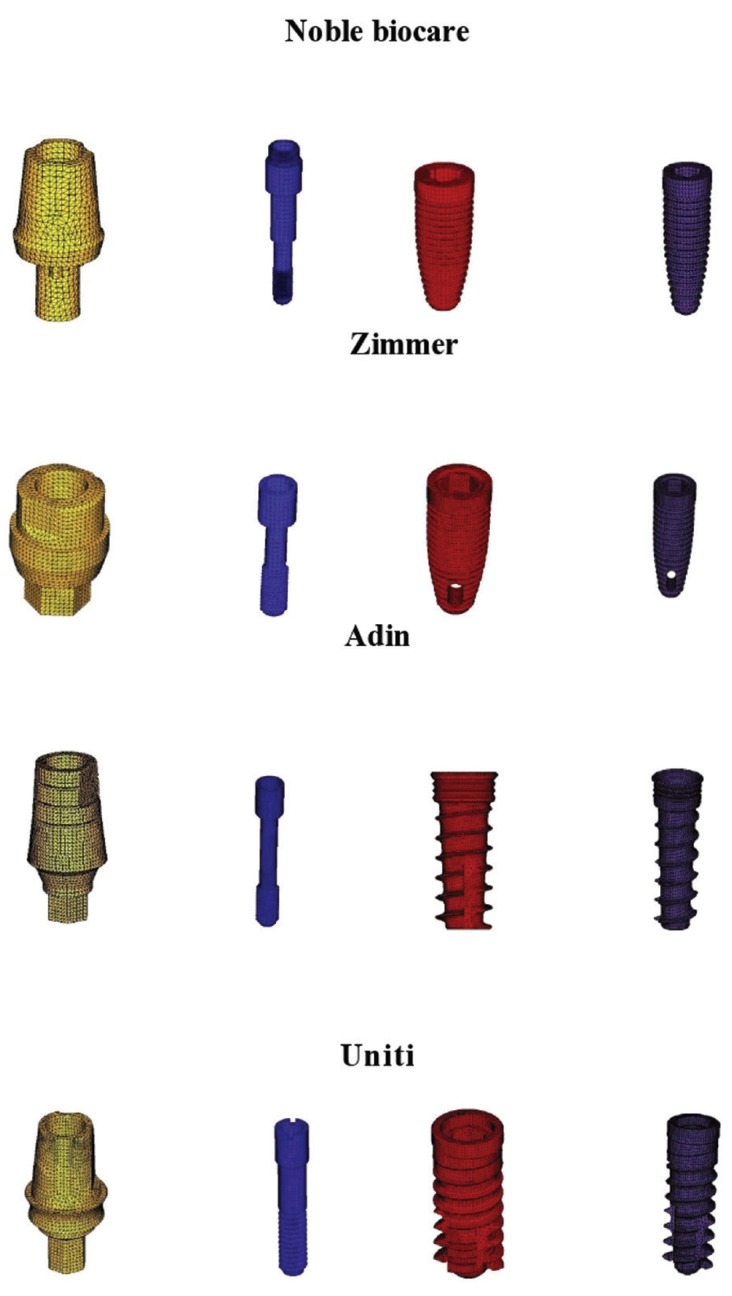
FEA models of Abutment, inscrew, implants.

**Figure 2:**
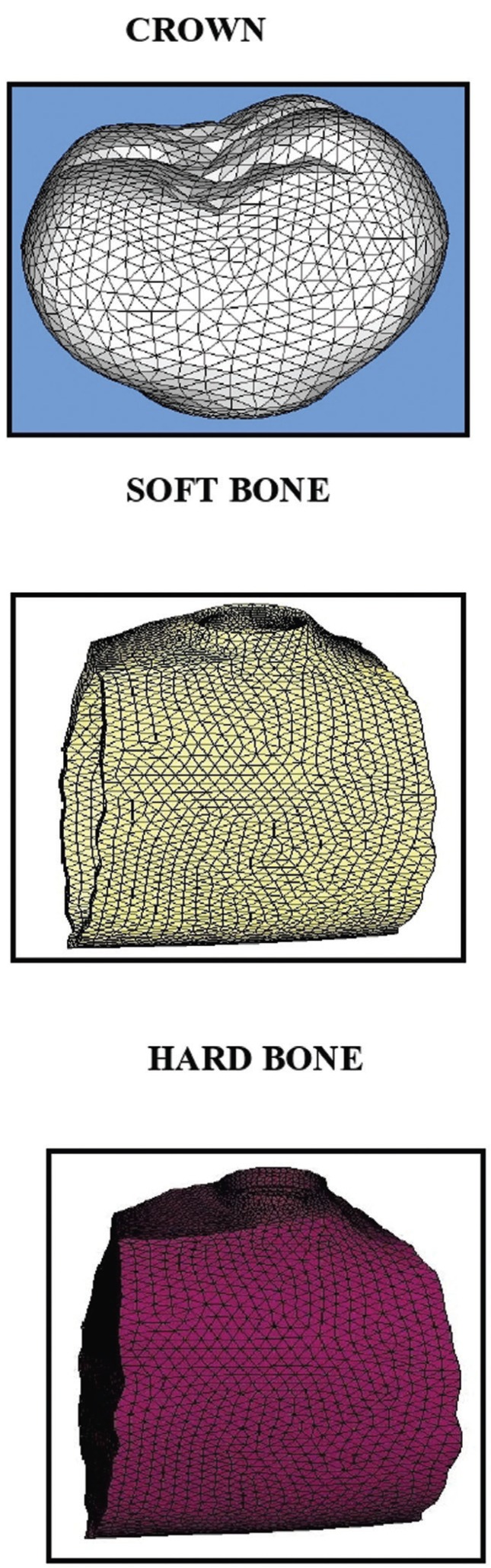
FEA models of crown soft bone and hard bone.

1. The geometric models of the Implant, inner screw, and abutment for all 8 designs were modeled using Solid Edge software by using reverse engineering technique (Creating the 3D CAD model by physically measuring and extracting the dimensions of the parts/components using precision tools and software)

2. The geometric model of the bone and crown was obtained from the CT scan

3. The geometric models (surface and line data) are then imported into Hypermesh software for meshing

4. The process of converting geometric model into finite element model is called meshing

5. Finite element model consist of nodes and elements (Figs. 1,2).

6. Assembled finite element model of the implants crown and bone is then imported into Ansys software for analysis

7. The material properties (young’s modulus and Poisson’s ratio) of crown, abutment, inner screw, implant, soft bone , cortical bone, and cement are entered in the pre-processing stage

8. The loads and boundary conditions mentioned above are applied in the solution stage

9. Solving stage: solving each load case separately( vertical, oblique and lateral)

10. Post-processing the results and capturing the displacement and von-misses stress contours of each individual parts in the system

11. pre-processing, solving and post-processing are three stages in ansys.

## Results

The stress distribution pattern between vertical lateral and oblique forces among all the samples used were analyzed using ANOVA and Turkey HSD test with a total sample size of 24 with 8 in each group. The mean, standard deviation, significance, and percentage of significance for the3 loading condition in all the components are given in the above table the results showed that in the soft bone, and hard bone there was 99.9% significance and 99% significance in inscrew using ANOVA. The multiple comparisons between vertical, lateral, and oblique forces was done using TURKEY HSD test the results showed a significance of 99% between lateral and oblique loads in the inscrew and 99.9% significance between the lateral and vertical, and lateral and oblique in soft bone and hard bone.

The stress distribution patterns between surface coated implants and non surface coated implants were analyzed using Independent sample test in vertical, oblique, and lateral load with a sample size of 4 in each group. The mean, standard deviation, significance, and percentage of significance for all the components between the surface coated implants (represented as S) and non surface coated implants (represented as Ns) are given in the  Table 1. The results showed that in the INSCREW and the full component there was 95% significance. There was a significance of 0.037 and 0.038 when equal variances was assumed and not assumed respectively for an INSCREW and in full components the significance values were 0.024 and 0.026 respectively.

**Table 1:**
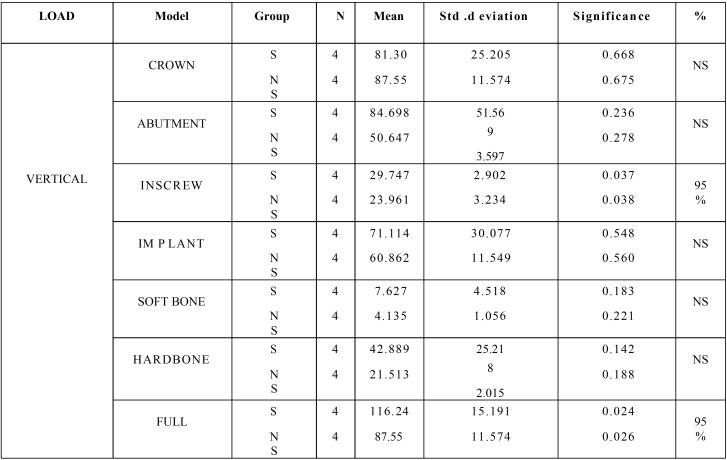
Statistical results between surface coated and non surface coated implants for vertical load.

The mean, standard deviation, significance, and percentage of significance for all the components between the surface coated implants (represented as S) and non surface coated implants (represented as Ns) are given in the  Table 2. The results showed 99% significance in the INSCREW and in the full component there was 95% significance. There was a significance of 0.002 and 0.006 when equal variances was assumed and not assumed respectively for an INSCREW and in full components the significance values were 0.039 and 0.076 respectively. The mean, standard deviation, significance, and percentage of significance for al l the components between the surface coated implants (represented as s) and nonsurface coated implants (represented as ns) are given in the  Table 3 the results showed that in the INSCREW and the full component there was 95% significance. There was a significance of 0.037 and 0.038 when equal variances was assumed and not assumed respectively for an INSCREW and in full components the significance values were 0.031 and 0.034 respectively.

**Table 2:**
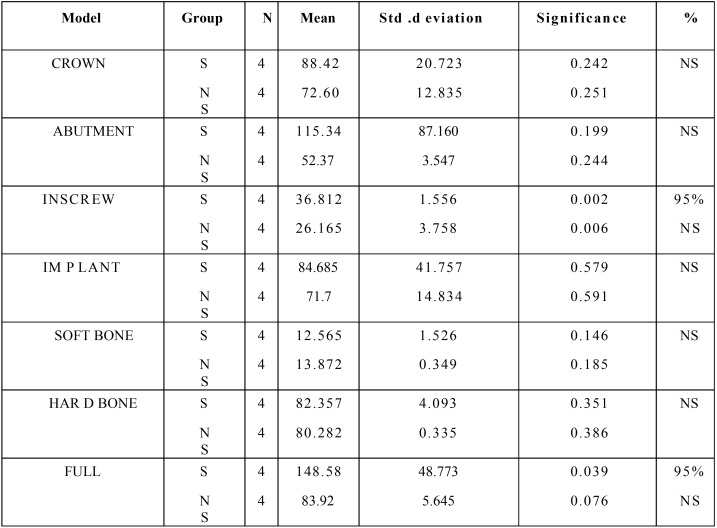
Statistical results between surface coated and non surface coated implants for lateral load.

**Table 3:**
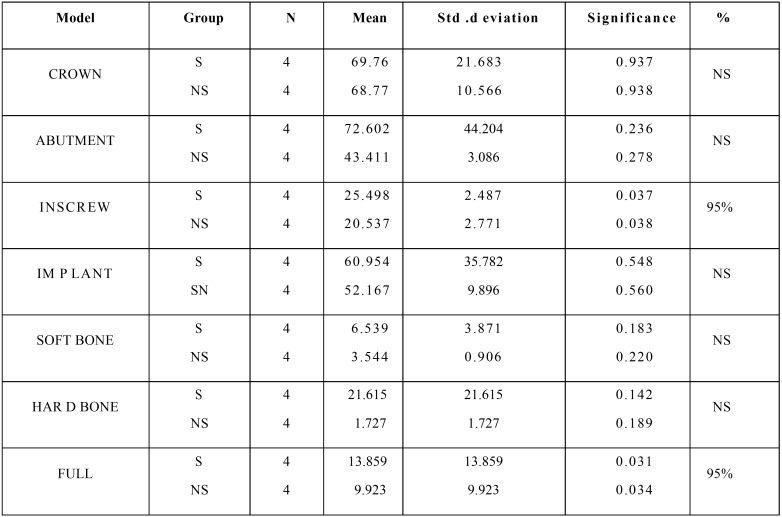
Statistical results between surface coated and non surface coated implants for oblique load.

## Discussion

FEA allows the estimation of the stress/strain state of extremely geometrically complex systems such as the dental implant–bone system. The more detailed the representation of its structural integrity, the more reliable the numerical results obtained. Thus, the prediction of dental implant success (9,10). In a two dimensional method it is not possible to study horizontal or oblique bite forces. Therefore it is not a valid representation of a clinical situation (11,12). To suit the aims of this study, a three dimensional finite element model was generated, which was well suited to study the true biomechanical behavior. However, as a limitation in their study, oguz eraslan and ozgur inan (13). when compared four different thread profiles analyzed implants that dint have threads at neck region. And they stated that Implants having threads at neck region are commercially available, and this configuration type can be compared at a further study. So commercial available implants were taken as samples in this study. The magnitude of stress is dependent on two variables: Force magnitude and cross sectional area over which the force is dissipated. The force magnitude can rarely be completely controlled, but the functional cross sectional area over which the force is distributed can be optimized by selecting an implant geometry that has been carefully designed to maximize functional area. An increase in functional area serves to decrease the magnitude of mechanical stress imposed on the prosthesis, implant, abutment and biologic tissues (12).

Abutment type has significant influence on the stress distribution because of different load transfer mechanisms and the differences in size of contact area between the abutment and implant Heoung jae *et al.* have concluded that the internal hex abutment generated the least von mises stress for all the loading condition so straight abutments with internal hex were used in this study (13). In Normal masticatory function, occlusal loads cover a range of values from 15 to 50 N so 3 different forces were used with different direction of application of force to stimulate the dynamic nature of the forces generated in the mouth (14,15).

The implant diameter and length are responsible for microstrains, stresses, and eventually for micromotions generated at the bone-implant (16-18). The literature contains no studies defining with clarity the relationship between implant length and success rates. It has been reported that increasing implant length affects success rates up to a limit, while other studies report that implant length does not significantly affect survival rates, yet other studies correlate short implants either with increased failures or with similar outcomes to those reported with longer implants (19-20). Renouard *et al.*, trying to explore the high failure rates of short implants, revealed that the surgical protocol used for short implant insertion did not include factors such as the evaluation of the bone quality and the implant surface. so surface coated commercially available tapered internal hex implants with different lengths were used in this study.

-Sites of maximum and minimum stress concentration

A consistent observation from all the models was concentration of maximum stress at the bone-implant interface at the level of neck of implant. This is in agreement with the findings of Hoshaw *et al.* who conducted studies on tibia bones of dogs under cyclic stresses and 3D FEA conditions. (21) Another Consistent observation is near absence of stress in the apical region of the implants. This supported the findings of Block *et al.* who demonstrated that the amount of bone directly in contact with the apical surface of a loaded implant was much less than that surrounding the remainder of the implant. From this observation it was concluded that the apical region of the implant within the cancellous bone had little stress-induced stimulation. (22) For vertical load the maximum stress concentration was on the crown except for zimmer implants which was on the abutment. For oblique load the maximum stress concentration was on the crown except for zimmer implants which was on the abutment. For lateral load the maximum stress concentration was on the crown for noble biocare and adin implant with 13mm length but was on the abutment for zimmer implant, the hard bone for uniti implant and at implant for adin implant with 10mm length.

-Stress distribution patterns between different lengths (10mm, 13mm) 

Vertical load

**Figure 3:**
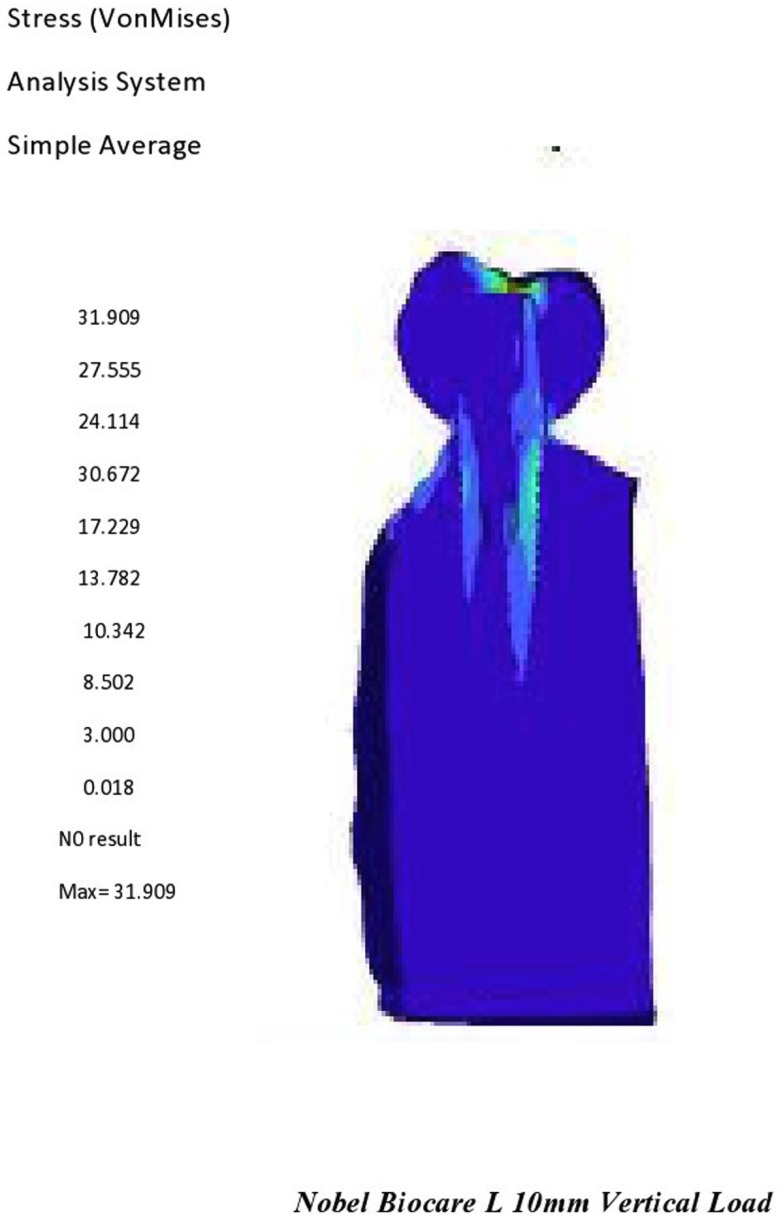
Full component of noble biocare 10mm length on vertical load of 30N.

There was a decrease in stress concentration at the crown for both the surface coated implants. Increase in stress concentration in abutment, Inscrew, and implant was observed in both the surface coated implants. Mixed results were found in relation to bone condition. There was a decrease in stress concentration at the implant and hard bone among the non surface coated implants. Increase in stress concentration was observed only in Inscrew for both the non surface coated implants. Mixed results were found in relation to crown, abutment, and soft bone (Fig. 3).

Oblique load

Among the surface coated implants there was a decrease in stress concentration in the crown. Among the surface coated implants there was an increase in stress concentration at the abutment, inscrew. And implant. Among the non surface coated implants there was a decrease in stress concentration within implant and hard bone. Among the non surface coated implants there was an increase in stress concentration at the inscrew. Mixed results were found in relation to crown, abutment, and soft bone.

Lateral load

There was a decrease in stress concentration at the crown for both the surface coated implants. Increase in stress concentration in abutment, Inscrew, and soft bone was observed in both the surface coated implants. Mixed results were found in relation to implant and hard bone. There was a decrease in stress concentration at the implant and hard bone for both the non surface coated implants. Mixed results were found in relation to crown, abutment, inscrew, and soft bone.

-Stress distribution patterns among all implants for vertical, lateral and oblique forces.

The multiple comparisons between vertical, lateral, and oblique forces was done using TURKEY HSD test the results showed a significance of 99% between lateral and oblique loads in the inscrew and 99.9% significance between the lateral and vertical, and lateral and oblique in soft bone and hard bone

-Stess distribution patterns between surface coated implants and non surface coated implants

Among surface coated and non-surface coated implants the pattern of stress distribution was almost similar except at the inscrew. The amount of significance for lateral load was 99% but was similar between vertical load and oblique load in an inscrew among surface coated and non surface coated implant groups. The pattern of stress distribution was almost similar between vertical and oblique loading condition But varied with lateral load between surface coated and non surface coated implants.. So there is no significant stress distribution variation at implants between surface coated and non surface coated implants.

## Conclusions

The conclusions of this computer study are limited to the assumptions involved in the construction of the

computer models. Within the scope of this study, the following observations were made.

1. The site of maximum stress concentration at the implant was always at the neck of the implant for all the 3 forces and all the 8 implants.

2. The minimum stress concentration was always at the soft bone for all the 3 forces and all the 8 implants.

3. As the length of the implant increased stress concentration had a tendency to increase at the abutment and Inscrew on all the 3 forces.

4. The stress distribution patterns between vertical, lateral, and oblique forces showed similarity in all components except in soft bone, hard bone and Inscrew.

5. The pattern of stress distribution was almost similar between vertical and oblique loading but varied with lateral load between surface coated implants and non surface coated implants.

6. Among the surface coated and non surface coated implants the pattern of stress distribution was almost similar except at the Inscrew Signifying that surface coating of implants had no significant role in stress distribution at the implant but had signifying stress distribution effect at the abutment screw.

-Future direction

Modeling of the bone– implant interface should incorporate the actual osseointegration contact area in cortical bone as well as the detailed trabecular bone contact pattern through the use of contact algorithms in FEA. Since there was significance at the inscrew, soft bone and hard bone. Future studies should aim at optimizing the design modification at the inscrew as the bone quality cannot be controlled by the manufactures.
